# Vitamin B_2_ as a virulence factor in *Pseudogymnoascus destructans* skin infection

**DOI:** 10.1038/srep33200

**Published:** 2016-09-13

**Authors:** Miroslav Flieger, Hana Bandouchova, Jan Cerny, Milada Chudíčková, Miroslav Kolarik, Veronika Kovacova, Natália Martínková, Petr Novák, Ondřej Šebesta, Eva Stodůlková, Jiri Pikula

**Affiliations:** 1 Laboratory of Fungal Genetics and Metabolism, Institute of Microbiology, Academy of Sciences of the Czech Republic, Prague, Czech Republic; 2 Department of Ecology and Diseases of Game, Fish and Bees, University of Veterinary and Pharmaceutical Sciences Brno, Czech Republic; 3 Department of Cell Biology, Faculty of Science, Charles University in Prague, Czech Republic; 4 Institute of Vertebrate Biology, Academy of Sciences of the Czech Republic, Brno, Czech Republic

## Abstract

Pathogenic and non-pathogenic related microorganisms differ in secondary metabolite production. Here we show that riboflavin overproduction by a fungal pathogen and its hyperaccumulation in affected host tissue exacerbates a skin infection to necrosis. In white-nose syndrome (WNS) skin lesions caused by *Pseudogymnoascus destructans*, maximum riboflavin concentrations reached up to 815 μg ml^−1^, indicating bioaccumulation and lack of excretion. We found that high riboflavin concentrations are cytotoxic under conditions specific for hibernation, affect bats’ primary fibroblasts and induce cell detachment, loss of mitochondrial membrane potential, polymerization of cortical actin, and cell necrosis. Our results explain molecular pathology of WNS, where a skin infection becomes fatal. Hyperaccumulation of vitamin B_2_ coupled with reduced metabolism and low tissue oxygen saturation during hibernation prevents removal of excess riboflavin in infected bats. Upon reperfusion, oxygen reacts with riboflavin resulting in dramatic pathology after arousal. While multiple molecules enable invasive infection, riboflavin-associated extensive necrosis likely contributes to pathophysiology and altered arousal pattern in infected bats. Bioaccumulation of a vitamin under natural infection represents a novel condition in a complex host-pathogen interplay.

Microorganisms produce metabolites to support their physiological functions and biotic interactions^1^. These may induce deleterious toxicity and contribute to microbial pathogenicity or play crucial roles as compounds vital for animal metabolism. Among these metabolites, riboflavin (vitamin B_2_) enables microbial intracellular survival and promotes virulence^2^,^3^,^4^. Although vertebrates cannot synthesize it, riboflavin is required for oxidoreduction metabolic processes^5^. It may also be toxic to cells exposed to light^6^; however, healthy, euthermic vertebrates avoid these adverse effects by excretion of its excess in urine.

Multiple molecules likely enable fungal invasive infection of hibernating bats with *Pseudogymnoascus destructans*^7^,^8^,^9^, a generalist pathogen and causative agent of white-nose syndrome (WNS)^10^,^11^,^12^,^13^,^14^,^15^,^16^. Being restricted to the skin, WNS is not induced by a systemic fungal infection, yet it resulted in population decline in six Nearctic bat species^17^.

Here we investigate molecular mechanisms of fungal pathogenicity that lead to tissue damage and potential detrimental physiological cascade in infected animals. We combine evidence from analytical chemistry, microbiology, pathology and cell biology to discover that accumulation of riboflavin within bat skin may act as a virulence factor of WNS. In fact, this finding links existing data and hypotheses on WNS^18^,^19^,^20^,^21^,^22^ by revealing a novel mechanism of pathophysiology, operating in both torpid and euthermic animals.

## Results

**Figure 1:**
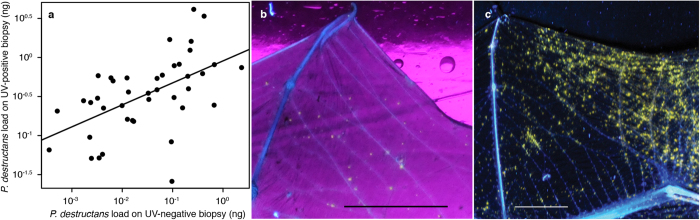
Ultraviolet fluorescence on bat wings corresponding with WNS lesions and increased *P. destructans* load. (**a**) Wing punch biopsies with UV fluorescent spots contain higher fungal loads than do paired spots without fluorescence (y = 0.294x + 0.015, *F*_1,52_ = 20.83, *p* < 0.001, *r*^2^ = 0.29). Fungal load was quantified from a wing punch biopsy using qPCR with TaqMan chemistry and dual probes^50^. (**b**) Transillumination of a wing membrane of Palearctic *Myotis dasycneme* shows yellow-orange spots at wing areas indicative of WNS lesions. (**c**) Transillumination of a wing membrane of Nearctic *Myotis lucifugus* showing WNS-associated yellow-orange fluorescence over extensive wing area. Image modified with permission from^15^ Scale bar −1 cm.

**Figure 2:**
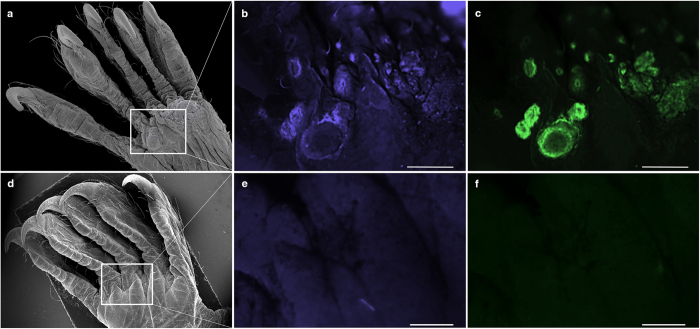
Visualization of fluorescent skin lesions in plantar surface of *M. myotis*. (**a**) Scanning electron microscopy shows circumscribed dermal nodules elevated over the skin’s surface. (**b**) Fluorescence microscopy, blue emission (UV excitation) showed fluorescent foci at sites with dermal nodules. (**c**) Fluorescence microscopy, green emission (blue excitation) showed fluorescent foci at sites with dermal nodules with slightly different pattern to (**b**). Fluorescence spectral variation in the WNS lesions indicates spatial distribution of secondary metabolites produced by *P. destructans* during progressing skin infection. (**d–f**) A control, euthermic bat captured in summer when bats heal from WNS had no nodular lesions on its plantar skin photographed with scanning electron microscopy (**d**), and with fluorescence microscopy using blue (**e**) and green (**f**) emission. Scale bar–500 μm.

The extent of skin damage due to invasive growth of *Pseudogymnoascus destructans* (Fig. 1a) is visible under ultraviolet (UV) light transillumination, documenting lower disease intensity in a Palearctic bat compared to WNS lesions over extensive wing area in a Nearctic bat (Fig. 1b,c). We define WNS lesions as wing membrane sites that fluoresced after illumination with 360 nm UV light as yellow-orange spots (Fig. 1) in cases where *P. destructans* was identified and confirmed with histopathology to invade living tissue of the bat. Whole-body screening of a *Myotis myotis* specimen using a stereo microscope revealed fluorescent foci both under UV and blue excitation (Fig. 2). Scanning electron microscopy revealed circumscribed dermal nodules elevated over the skin’s surface at sites with UV-confirmed WNS lesions (Fig. 2a). While fluorescent WNS spots contain 0.03–4.13 ng (median = 0.37 ng, *n* = 54) of *P. destructans* DNA per 7 mm^2^ of wing biopsy based on quantitative polymerase chain reaction (qPCR) (Fig. 1a), flying membrane without UV fluorescence had significantly lower fungal loads (paired *t*-test: *t*_53_ = −9.972, *p* < 0.001).

**Figure 3:**
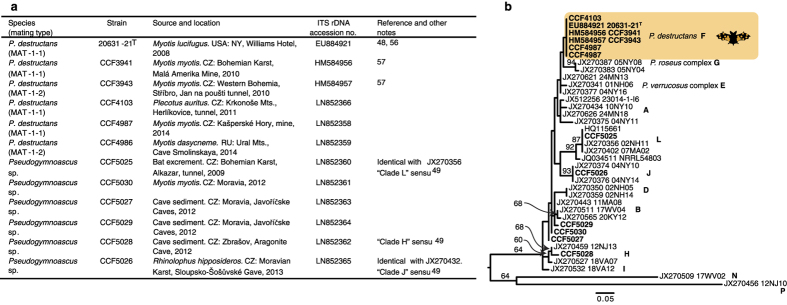
*Pseudogymnoascus* strains used in the study. (**a**) Table listing fungal strains’ identification and origin^53^,^54^. Two nonpathogenic *Pseudogymnoascus* strains were isolated from swab samples from bats negative for WNS on UV transillumination test and histopathology evaluation. CZ–Czech Republic, RU–Russia. (**b**) Maximum likelihood phylogenetic tree based on ITS rDNA sequences showing relatedness of 12 isolates used in this study (in bold). The closest blastn matches to sequences from our study were downloaded from NCBI GenBank. Sequence alignments were obtained using the MAFFT 7 G-INS-1 algorithm^55^. Maximum likelihood analyses^56^ were performed using a K2P + I substitution model chosen with an Akaike Information Criterion comparison^57^. Bootstrap support was obtained from 1,000 pseudoreplicates. Taxonomy was adopted from Minnis and Lindner^49^ and displayed with letters at the published clade representatives. Sequences with identical residues were added into the tree after analyses. Scale bar depicts substitutions per site.

**Figure 4:**
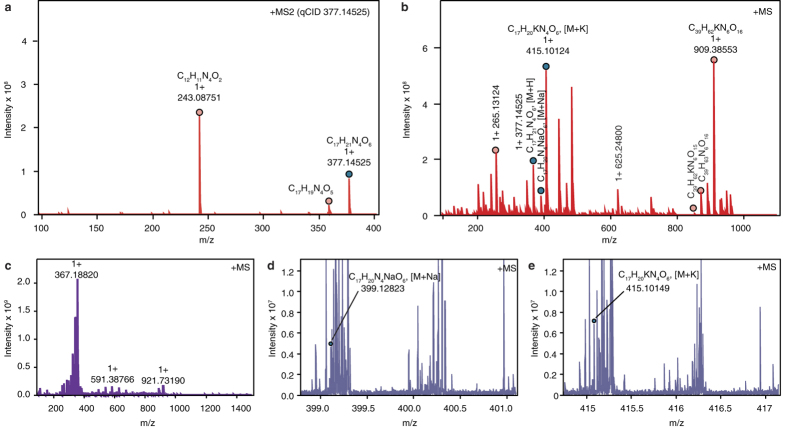
FTMS and MS/MS data for riboflavin. (**a**) MS/MS fragmentation of riboflavin. Lumichrome was detected with molecular mass at m/z 243.08751. (**b**) FTMS spectrum of the SPE extract of 8-week-old liquid cultivation medium of *P. destructans* strain 20631-21^T^. (**c**) FTMS spectrum for riboflavin in bat skin containing WNS lesions. Ultraviolet light guided biopsy from uropatagium of a WNS-positive *M. myotis* was homogenized with liquid nitrogen and prepared for the analysis in 40% MeOH/H_2_O. (**d,e**) Magnification of the FTMS spectrum for riboflavin from bat skin biopsy. Blue circles indicate riboflavin detection.

In search of fluorescent compounds among secondary metabolites of *P. destructans*, we investigated crude extract (CH_2_Cl_2_) of glucose yeast liquid cultivation medium of the strain *P. destructans* CCF3941 (see Fig. 3a for isolates details and Fig. 3b for their phylogenetic context) fractionated on a solid phase extraction (SPE) C 18 cartridge using a stepwise gradient of methanol in water. The fraction eluted with 80% methanol contained a pure yellowish compound. Using UV and Fourier transform mass spectrometry (FTMS), we identified the compound as lumichrome (molecular mass [M + H] at *m/z* 243.08751 with elemental composition C_12_H_11_N_4_O_2_, Fig. 4a). Lumichrome is a degradation product of riboflavin. To prevent riboflavin modification (formation of lumichrome as an experimental artifact), the liquid cultivation medium was directly loaded on the SPE C 18 cartridge and eluted with methanol. Methanol eluate was directly analyzed by FTMS and tandem mass spectrometry (MS/MS). The determined molecular mass [M + H] at *m/z* 377.14525 with elemental composition C_17_H_21_N_4_O_6_ signified riboflavin (Fig. 4b). Likewise, riboflavin was directly determined with FTMS in a 40% methanol fraction from a homogenized bat skin biopsy that contained WNS lesions (Fig. 4c, and see Fig. 4d,e for FTMS spectrum magnification that includes riboflavin).

The crude extract fraction from *P. destructans* liquid cultivation medium eluted with 50% MeOH also contained a cinnamon-colored compound. Analysis with FTMS and MS/MS identified the compound as the siderophore [Fe^3+^] triacetylfusarinine C (data not shown).

**Figure 5:**
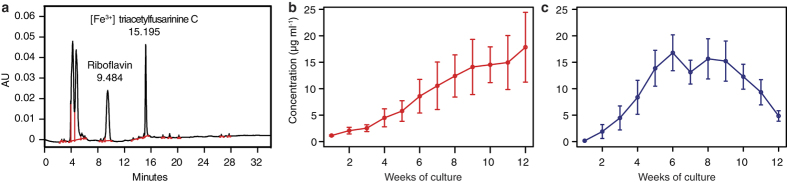
Production of riboflavin in *Pseudogymnoascus destructans*. (**a**) HPLC chromatogram of SPE C18 extract from liquid cultivation medium of *P. destructans* strain 20631-21^T^ (see Fig. 6a,b for medium and blank controls). (**b**) Average production curve (±standard error) of riboflavin in six strains of *P. destructans* (Fig. 3) shows continuous metabolite accumulation. (**c**) Average production curve of [Fe^3+^] triacetylfusarinine C in six strains of *P. destructans* peaks after 6 to 8 weeks of culture. The concentration of the metabolite was corrected for biomass content in the fermentation medium.

**Figure 6:**
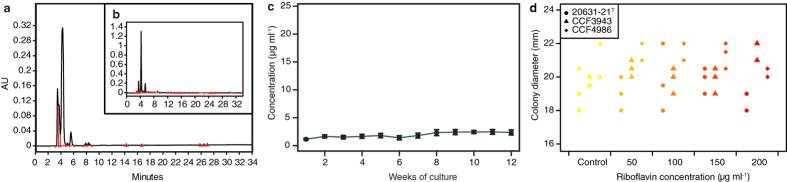
Production and tolerance of riboflavin in *Pseudogymnoascus* fungi. (**a**) HPLC chromatogram of SPE C18 extract from liquid cultivation medium of *Pseudogymnoascus* sp. strain CCF5026. (**b**) Blank liquid cultivation medium HPLC chromatogram. (**c**) Average production curves (±standard error) of riboflavin in non-pathogenic *Pseudogymnoascus* strains. The concentration of the metabolite was corrected for biomass content in the fermentation medium. (**d**) Tolerance of *P. destructans* strains to riboflavin in concentrations ranging from 0 to 200 μg ml^−1^. Riboflavin was added after autoclaving the medium containing glucose (20 g l^−1^), yeast extract (5 g l^−1^), and agar (20 g l^−1^). Colony diameter was measured after 49 days. Three replicates were used in each experiment, and their values may overlap.

We used reversed-phase high-performance liquid chromatography (HPLC) to determine presence of the main extracellular metabolites (i.e., riboflavin and [Fe^3+^] triacetylfusarinine C) in stationary cultivated liquid cultivation medium (Fig. 5a). All 12 tested *Pseudogymnoascus* isolates (Fig. 3) produced riboflavin but differed in those quantities. While *P. destructans* strains produced riboflavin in concentrations up to 37 μg ml^−1^ after 12 weeks of culture (Fig. 5b), nonpathogenic *Pseudogymnoascus* spp. strains produced a maximum of 4 μg ml^−1^ in the same time (Fig. 6c). The second main metabolite, [Fe^3+^] triacetylfusarinine C, was produced only by pathogenic strains and its maximum concentration (26 μg ml^−1^) was reached between 6 and 8 weeks of cultivation (Fig. 5c). Riboflavin concentrations ranging from 25 to 200 μg ml^−1^ did not inhibit growth of *P. destructans* compared to the control (Fig. 6d).

**Figure 7:**
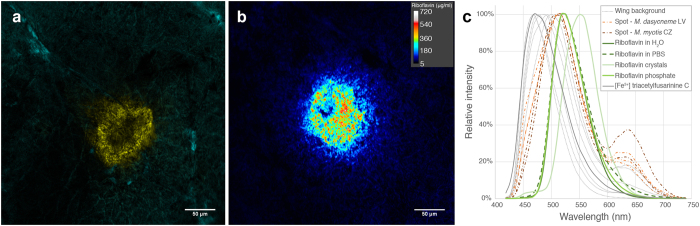
Quantification of riboflavin in a WNS lesion. (**a**) Fluorescence of skin lesion excited with UV light (405 nm). (**b**) Quantification of riboflavin content in skin lesion based on fluorescence signal. (**c**) Emission spectra profiles (lambda scan) of standard chemicals, wing background, and yellow-orange fluorescence regions of skin lesions observed in two bat specimens. CZ–Czech Republic, LV–Latvia, PBS–phosphate-buffered saline.

The secondary metabolite production of *P. destructans* varies in time (Fig. 5b) and across tissue (Fig. 2, Supplementary Video 1). To estimate riboflavin concentration in a naturally infected bat wing, we used the lambda-scanning feature of a confocal microscope. UV and blue excitation colour channels revealed a nonhomogeneous distribution of fluorescence patterns across the wing (Fig. 7). The background emission spectrum of the bat wing membrane peaked between 460 and 510 nm (Fig. 7c). The WNS lesion emission spectrum contained two characteristic peaks, with maxima at 510–518 nm and 633–641 nm. The emission spectrum of riboflavin dissolved in phosphate buffered saline mimicking physiological pH and osmolarity peaked at 513 nm. It constituted a major contribution to the fluorescence spectrum of the first peak in WNS lesions excited by the 405 nm laser. [Fe^3+^] triacetylfusarinine C, previously identified fluorescent compound associated with WNS^7^, exhibited an emission spectrum peak at 467 nm (Fig. 7c). This differed from the two peaks of the WNS lesions, thus indicating a minor contribution of [Fe^3+^] triacetylfusarinine C to the WNS lesions’ fluorescence. The WNS lesions’ second peak at 633 nm corresponds to another putative molecular component. We unmixed and separated two spectral curves, wing background and WNS spot-derived channels, to estimate the local concentration of riboflavin and its metabolites in the tissue (Fig. 7b). The mean riboflavin concentration per WNS lesion ranged from 54 to 141 μg ml^−1^ (median = 86, *n*_spots_ = 12, *n*_bats_ = 4), but local deposits fluoresced with maximum intensity up to 815 μg ml^−1^ (median = 200) (Supplementary Fig. S1).

**Figure 8:**
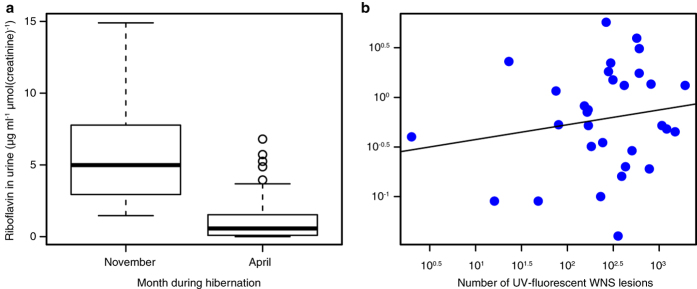
Riboflavin concentration in bat urine. (**a**) Time variation of riboflavin concentration in *M. myotis* urine between early hibernation (November, *n* = 36) and late hibernation (April, *n* = 64). (**b**) Relationship of riboflavin in bat urine and sum of WNS lesions detected during late hibernation by UV transillumination of both wings (*n* = 31). Regression of log-transformed variables was not significant (*P* = 0.34) and Pearson’s correlation was low (*r* = 0.17). Riboflavin concentration in bat urine was standardized to 1 μmol of creatinine to account for density of the urine sample.

To examine riboflavin urinary excretion in bats, we measured urine concentrations of riboflavin and creatinine with HPLC. Riboflavin concentration in bat urine was standardized to 1 μmol of creatinine to account for density of the urine sample. Lower urinary excretion of riboflavin during late hibernation (Wilcoxon rank test: *W* = 153, *p* < 0.001, *n* = 100) signified metabolite hyperaccumulation in skin. Urinary excretion of riboflavin did not correlate with infection intensity in late hibernation as quantified with the number of UV fluorescent lesions on bat wings (Pearson's *r* = 0.17, *P* = 0.345, *n* = 31; Fig. 8).

**Figure 9:**
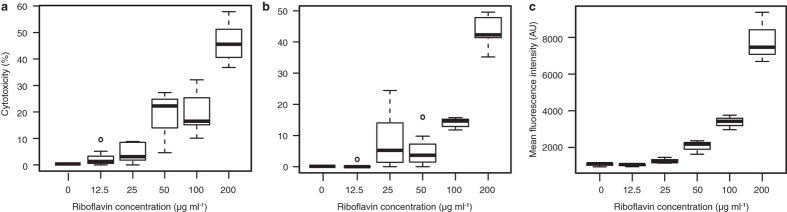
Cytotoxicity of riboflavin on primary bat fibroblasts, mimicking skin exposure to different temperature and light conditions during hibernation. (**a**) Cytotoxicity after 24 h of exposure to riboflavin at 8 °C, representing temperature during torpor. Experiment performed in septuplicate with 15,000 cells per replicate. (**b**) Cytotoxicity after 24 h of exposure to riboflavin at 37 °C, i.e. bat temperature during arousal from torpor. Experiment performed in septuplicate with 15,000 cells per replicate. (**c**) Reactive oxygen species (ROS) measured by fluorescence activated cell sorting for ROS sensor positivity. Cells were grown in biological triplicates to 50% confluency, incubated with riboflavin for 2 h, then illuminated in the presence of ROS sensor for 30 min at approximately 1/5 of daylight intensity. Number of cells evaluated using flow cytometry per each experiment ranged from 11,992 to 18,218.

We treated primary bat skin fibroblasts with a series of riboflavin concentrations (0–200 μg ml^−1^). Measuring lactate dehydrogenase activity indicative of cell death, riboflavin proved to be cytotoxic in a dose-dependent manner under conditions simulating hibernation torpor (darkness, 8 °C) from 12.5 μg ml^−1^ and simulating periodic arousals (darkness, 37 °C) from 25 μg ml^−1^ (Fig. 9a,b). To mimic the consequences of bat emergence from hibernacula prior to sunset, we approximated that bats might be exposed to reduced daylight intensities for a limited time. The cells exposed to 110 μmol of photons m^−2^ s^−1^ for 30 minutes, treated with riboflavin, demonstrated a rapid onset of oxidative stress from 25 μg ml^−1^ on flow cytometry (Fig. 9c).

**Figure 10:**
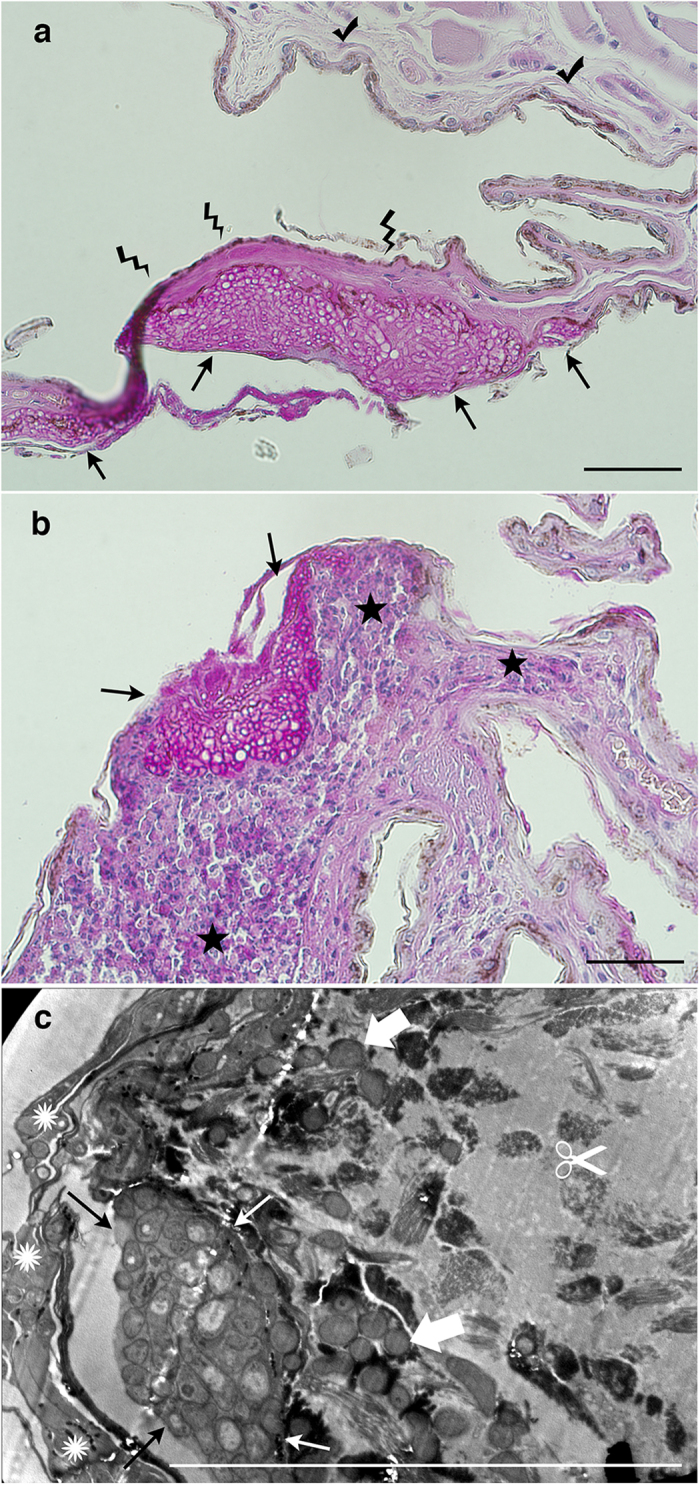
Histopathology of WNS lesions. (**a**) *M. daubentonii*, necrotic wing membrane tissue next to a cupping erosion packed with *P. destructans* hyphae (periodic acid–Schiff stain). (**b**) *M. dasycneme*, sequestration of WNS lesion in skin by intense neutrophilic infiltration (periodic acid–Schiff stain). (**c**) *M. myotis*, invasive skin infection by *P. destructans* hyphae and tissue necrotic derangement (transmission electron micrograph). Arrow: cupping erosion; lightning bolt: loss of staining pattern in necrotic tissue; checkmark: intact tissue; star: inflammatory infiltration with neutrophils sequestering the cupping erosion; sun: epidermal surface colonization by the fungus; block arrow: dermal invasion by the fungus; scissors: coarsely granular amorphous material and dermal edema, indistinct outlines of individual bat tissue cells, and derangement of elastic fibers. Scale bar–50 μm.

We visualized integrity and dynamics of three cellular compartments: mitochondria as a reflection of energy metabolism, the actin cytoskeleton as an indicator of overall plasma membrane shape and adhesion, and the nucleus as an indicator of cell cycle dynamics or apoptosis. Treatment with a riboflavin concentration of 200 μg ml^−1^ resulted in overall pathology (Supplementary Fig. S2). Fibroblasts began to detach after approximately 12 h. Mitochondria shape was altered to vesicle-like structures. Gradual decrease in mitochondrial fluorescence intensity indicated the loss of membrane potential and, therefore, depletion of adenosine triphosphate in bat fibroblasts. The probe for polymerized actin revealed a similar effect of high riboflavin concentrations (100 and 200 μg ml^−1^), indicating robust polymerization of cortical actin. No signs of apoptosis (fragmentation of nuclei, formation of apoptotic bodies) were detected visually. Instead, nuclear contraction indicating necrosis was typical for cells treated with a 200 μg ml^−1^ concentration of riboflavin (Supplementary Fig. S2), similar to necrosis in natural infections (Fig. 10a). Tissue necrosis in the vicinity of a WNS lesion manifested on histopathology as a loss of tissue structure and stainability (Fig. 10a,c). While no immune response was present around the necrotic tissue in some bats (Fig. 10a), other cases exhibited fungal sequestration by inflammatory infiltration with neutrophils (Fig. 10b).

## Discussion

During hibernation, bats enter a hypometabolic and hypoper-fusion state, when tissues exhibit low oxygen levels and biomolecules are slowly excreted or metabolised. Tissues of hibernators show natural resistance to the hypoperfusion–reperfusion experienced in repeated cycles of torpor and arousal during hibernation^23^. At the site of skin infection by *P. destructans*, hypoperfusion promotes accumulation of fungal metabolites, including riboflavin.

Intercontinental differences in disease outcome are dependent on infection intensity that differs between taxa^15^,^24^ (Fig. 1), not on pathogenic mechanisms exerted by the infectious agent. *Pseudogymnoascus destructans* strains from Europe, Asia and North America produce riboflavin, which accumulates in liquid cultivation medium and in skin lesions produced by the fungus (Fig. 4). We tested a full range of riboflavin concentrations to its upper solubility limit at 200 μg ml^−1^. These concentrations are likely encountered by cells as the fungus secretes up to 37 μg ml^−1^ of riboflavin after 12 weeks of culture (Fig. 6) and its mean concentration in WNS lesions ranges between 54 to 141 μg ml^−1^ with maxima at hundreds μg ml^−1^ (Supplementary Fig. S1). Focal concentrations or deposits, albeit possibly underestimated due to possible effects of fluorescence quenching, are beyond concentrations that are cytotoxic to cells in hibernation-like conditions (Fig. 9a,b) and induce oxidative stress in conditions simulating emergence from hibernacula (Fig. 9c).

Spectroscopic fingerprints allow differentiation of autofluorescent microorganisms^25^. The photochemical quality of riboflavin^26^ and its hyperaccumulation within the infected skin tissue is responsible for the distinctive orange-yellow fluorescence in UV light used to identify bats positive for WNS^15^. Various dermatophytes fluoresce (including representatives from *Trichophyton* and *Microsporum*), but most do not grow at low temperatures typical for microclimate in hibernacula. Although *Trichophyton* and *Microsporum* and other geophilous dermatophytes occur frequently in the hibernacula sediment, these fungi were missing on the skin of hibernating bats, even if the selective media were used^27^,^28^,^29^,^30^. There is currently no support that other microorganisms with yellow to orange fluorescence co-occur on bats with *P. destructans* at significant levels. Additionally, in contrast with conventional dermatophytes, UV fluorescence of *P. destructans* infection is not associated with fungal growth restricted to the skin surface. It is elicited only after invasive growth through and replacement of living tissues with cup-like erosions pathognomonic for WNS that are packed with fungal hyphae of *P. destructans*. This characteristic makes UV-guided biopsy a highly successful diagnostic tool with sensitivity from 95.5 to 98.8% and 100% specificity for WNS^15^. Infections of hibernating bats similar in appearance to WNS caused by a dermatophyte *Trichophyton redellii* were not observed to fluoresce like *P. destructans*-produced lesions^31^. *Trichophyton redellii* is easily cultivable and identifiable and was not found during our surveys.

Fluorescence spectral pattern that appears after *P. destructans* skin invasion indicates that other molecules are responsible for breaching the protective skin barrier and utilizing resources from living tissues. *Pseudogymnoascus destructans* secretes proteolytic and hydrolytic enzymes^8^,^32^,^33^, putatively facilitating skin invasion. Requirement for iron in microorganisms invading host tissues^34^ might explain *P. destructans* co-secretion of riboflavin and [Fe^3+^] triacetylfusarinine C. Microorganismal iron uptake, that siderophores provide, requires reduction of Fe^3+^ to Fe^2+^, for which flavins donate an electron^35^. Our results show that while riboflavin linearly accumulates in liquid cultivation medium, [Fe^3+^] triacetylfusarinine C production peaks after six weeks, which might reflect in a WNS lesion as minor contribution of the siderophore to UV fluorescence.

Cell cultures derived from tissues of bat species at risk may provide a cutting-edge tool to study innate responses to WNS^36^. To evaluate biological activity of riboflavin concentrations observed in WNS skin lesions, we considered the fact that riboflavin produces free radicals upon contact with oxygen and exposure to light^5^,^6^. Such chemical reactions lead to oxidative injury of tissues, to modulation of cell signalling to apoptosis, or to necrosis^37^,^38^. Derangement of connective tissue cells characterizes a virulent *P. destructans* infection^10^ (Fig. 10c). Gradual decrease in mitochondrial fluorescence intensity indicated the loss of membrane potential and, therefore, depletion of adenosine triphosphate in bat fibroblasts.

Natural, invasive *P. destructans* infection of hibernating bats showed variability of histopathological findings (Fig. 10). The differential pathology likely represents a time series, with necrosis during hibernation torpor-arousal cycles and inflammation in early post-hibernation period^19^,^39^.

Hibernation affects both innate and adaptive immune responses to pathogens and increases host infection risk^39^,^40^. Evolutionarily conserved innate-like mucosal-associated invariant T cells use metabolites of the riboflavin biosynthetic pathway to detect microbial infection^41^, produce pro-inflammatory cytokines, and are cytotoxic^42^. The riboflavin molecular signature of infection by *P. destructans* associated with extensive wing area damage may thus trigger the intense skin pathology (Fig. 10) observed in bats during the early euthermic post-emergent season^19^. We conclude that with long duration of hibernation there is increased riboflavin accumulation, driven by suitable nutritional sources from infected skin and hypoperfusion of host tissues during torpor. High riboflavin concentrations damage skin, likely leading to an intensified arousal pattern, fat reserves depletion, and death. Photosensitizing properties of riboflavin might further contribute to the intense skin pathology observed in bats emerging from hibernacula during daylight^15^,^43^. The final disease outcome might be dependent on the total wing area damaged by *P. destructans* infection, which is larger in the Nearctic^15^,^44^ (Fig. 1c), where bats with WNS experience mass mortality^45^, than in the Palearctic (Fig. 1b), where they show tolerance to the disease^24^. Tissue damage from the hyperaccumulation of riboflavin and its oxidation at reperfusion proposed here illuminates disease progression in the multi-stage WNS model^21^ with a novel molecular mechanism. Considering the pathological effects of high concentrations of riboflavin within infected tissues, we suggest silencing or downregulation of genes from the endogenous biosynthetic pathway of riboflavin as potential targets for disease management^46^,^47^.

## Methods

### Animal sampling

Nonlethal sampling of wing membrane biopsies was performed in accordance with Czech Law No. 114/1992 on Nature and Landscape Protection, based on permits 01662/MK/2012S/00775/MK/2012, 866/JS/2012 and 00356/KK/2008/AOPK issued by the Agency for Nature Conservation and Landscape Protection of the Czech Republic. Experimental procedures were approved by the Ethical Committee of the Academy of Sciences of the Czech Republic (No. 169/2011). Sampling in Latvia was approved by the Nature Conservation Agency (Permit No. 3.15/146/2014-N) and in Russia by the Institute of Plant and Animal Ecology, Ural Division of the Russian Academy of Sciences (No. 16353–2115/325). The authors are authorized to handle free-living bats according to Certificate of Competence No. CZ01341 (§17, Act No. 246/1992 Coll.) and a permit approved by the Latvian Nature Conservation Agency (No. 05/2014).

Animals used in this study were sampled during 2014 and 2015 in accordance with the approved guidelines. To derive primary bat skin fibroblasts, a healthy bat (*M. myotis*) was captured during autumn swarming in the Czech Republic. Two 4-mm punch biopsies (Kruuse, Langeskov, Denmark) from each wing membrane were collected prior to releasing the bat. Samples for transmission or scanning electron (TEM or SEM) and confocal microscopy were cadavers without gross and histopathological signs of decomposition (e.g. epidermal/dermal separation and hair detachment, dermal degeneration and disintegration) and biopsies collected in hibernacula in the Czech Republic (*M. myotis*, *n* = 3) and Latvia (*M. dasycneme*, *n* = 1). We used samples where antemortem origin of fungal-induced tissue pathology was witnessed by neighbouring areas of normal skin histology within the same sample (cf. Fig. 10a,b). The cadavers were processed for experiments directly after transport to the laboratory (SEM and confocal microscopy), and UV fluorescent spots were recorded and biopsied for further analyses (confocal microscopy, FTMS, MS/MS, histopathology, TEM). Biopsies were stored at −80 °C (confocal microscopy, FTMS, MS/MS) or in liquid medium (histopathology, TEM). Live bats for fungal load quantification, urine collection and histopathology were handled so as to minimize stress and duration of sampling procedures between capture and release at the site. Bat’s right wing was extended on a sterilized glass slide over a UV lamp and two areas were biopsied: an area without discernible yellow-orange fluorescence (designated as UV-negative throughout the paper) and an area with UV fluorescence characteristic of WNS lesions (called UV-positive in the text). Biopsy punches were taken from bats in the Czech Republic and Latvia prior to their emergence^24^. Urine samples were collected with a pipette into amber light protection tubes from bats urinating during arousal initiated by handling.

### Fungal strains

Six strains of *Pseudogymnoascus destructans* representing various countries of origin (USA, Czech Republic, Russia) and mating types (MAT1-1-1, MAT1-1-2) were selected to study fungal extracellular metabolites. Six additional strains of four other *Pseudogymnoascus* spp. nonpathogenic to bats but living in their hibernacula were used for comparison (Fig. 3). All strains were characterized based on internal transcribed spacer (ITS) rDNA sequences and mating type idiomorphs according to Palmer *et al*.^48^. *Pseudogymnoascus* spp. isolates not belonging to *P. destructans* were compared to those published previously^49^, and their relatedness was demonstrated using a maximum likelihood phylogeny (Fig. 3).

### Cultivation conditions

Stock cultures of all monosporic strains were maintained on malt agar slants (malt extract 20 g l^−1^, agar 20 g l^−1^, pH adjusted to 6.5 by NaOH). Cultivation was performed on a glucose (20 g l^−1^) yeast (5 g l^−1^) extract liquid medium adjusted to pH 6.5. Stationary surface cultivations were carried out in 500 ml Erlenmeyer flasks containing 50 ml of the medium for 3 months at 8 °C in darkness.

### UV transillumination

The bat wing membrane was extended over a Wood’s lamp (366 nm; BLAK-RAY Model UVL-56, San Gabriel, CA, USA) and photographed with a Nikon D80 digital SLR camera (in cave: ISO 1000, f-stop 18, shutter speed 1.6–4 s; in dark room: ISO 400, f-stop 4–5, shutter speed 0.25–0.4 s) mounted on a tripod. The camera lens was directed perpendicular to the wing.

### Visualization techniques

Whole-body imaging was performed on a *M. myotis* specimen using a Zeiss Axio Zoom.V16 motorized fluorescent stereo microscope (Carl Zeiss Microscopy GmbH, Jena, Germany; UV, blue and green excitation light). Tissues from a dead animal with strong fluorescence signal corresponding to the presence of WNS lesions were excised (namely the flying membrane and pelvic limb), fixed (3.7% paraformaldehyde in PBS, 24 h, 4 °C), dehydrated via ethanol solutions, transferred to acetone, and processed for SEM applications (Bal-Tec CPD 030 critical point drier and Bal-Tec SCD 050 sputter coater). Visualization was performed using a JEOL JSM-6390 LV scanning electron microscope (JEOL USA, Peabody, MA, USA).

### Fungal load quantification

Wing punch biopsy samples were collected directly into tissue lysis buffer with proteinase K (DNeasy Blood & Tissue Kit, Qiagen, Halden, Germany) and isolated within 10 h of sampling according to the manufacturer’s protocol. Fungal load in the samples was estimated using a qPCR method based on TaqMan chemistry (Life Technologies, Foster City, CA, USA) and using dual probes for *P. destructans* and *Pseudogymnoascus* sp. detection^50^.

### Extraction and fractionation

Fermentation broth of the strain *P. destructans* CCF3941 was centrifuged and extracted three times with an equal volume of CH_2_Cl_2_. Pooled extracts were dried over anhydrous Na_2_SO_4_, filtered, then evaporated to dryness under reduced pressure. The crude extract (23.1 mg) diluted in MeOH (0.2 ml) was loaded into a 20 g SPE C18 cartridge (Phenomenex, Chromservis, Prague, Czech Republic), rinsed with water, then eluted stepwise with H_2_O/MeOH gradient (20%, 30%, 40%, 50%, 80%, 100% [V/V]). The fractions were evaporated to dryness and reconstituted in MeOH. The content of the fractions was checked using HPLC. The fraction eluted with 80% MeOH contained a pure yellowish compound and the fraction with 50% MeOH contained a cinnamon-colored compound, which were further analyzed using UV and FTMS.

### Solid phase extraction (SPE)

Fermentation broths of all tested strains (2 ml) and riboflavin standard solutions (Sigma, Steinheim, Germany) in water were loaded into a 0.5 g SPE C18 cartridge (Phenomenex, Chromservis, Prague, Czech Republic), conditioned with MeOH (2 ml), and equilibrated with H_2_O (2 ml). The cartridge was rinsed with water (5 ml) and eluted with MeOH (2 ml). Prior to HPLC analyses, the collected MeOH fractions were stored at −20 °C in darkness. All experiments were done in triplicates under conditions of reduced light.

### HPLC

The HPLC system consisted of a pump equipped with a 600E system controller, 717 autosampler, and 2487 dual UV detector (Waters, Milford, MA, USA). The data were processed using Empower 2 software. Water containing mobile phases had been filtered through a 0.22 μm GS filter (Millipore, Billerica, MA, USA) and degassed in an ultrasonic bath for 10 min before use.

A Gemini 5 μm C18 column (250 × 4.6 mm, Phenomenex, Torrance, CA, USA) with a guard column was used for the analysis. The mobile phase consisted of 5% MeOH in H_2_O and MeOH. Gradient elution started at 30% MeOH (0 min), increasing linearly to 100% MeOH within 20 min, at a flow rate of 1.0 ml/min. UV detection was performed at 260 and 350 nm.

For calibration experiments and quantitative determination of riboflavin, standard solutions of riboflavin were prepared in H_2_O at final concentrations of 1.25, 2.5, 5, 10, and 25 μg ml^−1^ (injected in triplicate). The calibration graph was constructed by plotting the integrated peak areas of individual compounds versus concentration. The linear regression equation y = 39081x–10630 with correlation (*r*) 0.999 and determination (*r*^2^) 0.999 coefficients were obtained for the HPLC procedure.

Samples of bat urine were diluted in water 20 times and injected directly. Riboflavin and creatinine were quantified using equation y = 43518x + 3292 (*r* = 0.999, *r*^2^ > 0.999) for riboflavin and y = 29803x + 50742 (*r* = 0.999, *r*^2^ = 0.999) for creatinine.

### Riboflavin recovery determination

Riboflavin recovery was determined at three different concentrations (6.25, 12.5, and 25 μg ml^−1^) and calculated by comparing the peak areas of the analytes in the extract from the spiked liquid cultivation medium and corresponding standard solutions in H_2_O (three replicates for each concentration). The recovery value of 98.54 ± 2.09% was determined for all concentrations tested.

### Mass spectrometry

Samples were measured on a commercial 12T solariX FTMS instrument (Bruker Daltonics, Billerica, MA, USA) equipped with an ESI/MALDI ion source and ParaCell. The analysis was performed using electrospray ionization (ESI) and the spectra were acquired in positive ion mode. The cell was opened for 0.75 ms, accumulation time was set at 0.1 s for the MS experiment (0.6 s for the MS/MS experiment), and one experiment (consisting of the average of four spectra) was collected per sample. After the MS experiment, one MS/MS experiment was performed for the ion of interest. The isolation window was set at 3 a.m.u. and the collision energy was kept at −16V. The size of the acquisition data set was set to 2M points with the mass range starting at *m/z* 100 a.m.u., resulting in a resolution of 350,000 at *m/z* 400. One μl of the sample was dissolved in MeOH/H_2_O (50%) and introduced into the mass spectrometer by direct infusion into the electrospray ion source. The instrument was externally calibrated using singly charged NaTFA clusters, resulting in sub-ppm accuracy. The spectra were apodized using sine apodization with one zero filling. Data were processed using Data Analysis 4.2 software (Bruker Daltonics), and possible elemental compositions were calculated using Smart Formula calculator.

### Confocal microscopy

Flying membrane samples were analyzed using a Leica TCS SP2 laser scanning confocal microscope (Leica Microsystems GmbH, Wetzlar, Germany) with a 405 nm diode laser for excitation and a 10x/0.4 objective. Lambda scans were performed using a 20 nm spectral window shifted continuously from 400 nm to 750 nm through the spectrum in 40 steps, thus yielding an 8.25 nm step. The obtained lambda stacks were analyzed with Leica LCS (Leica Microsystems GmbH) software and FIJI^51^. The calibration curve of riboflavin solutions (100, 80, and 50 μg ml^−1^) was analyzed in a focused drop on a cover slip under the same conditions as those of the lambda scans of the flying membranes. Linear curve fittings were computed in FIJI through the highest spectral points, yielding the linear regression equation y = 2.80777x + 4.68651, *r*^2^ > 0.999. Spectral unmixing of the natural background and fungal lesion spot spectrum were done in LCS software. The spectrally unmixed channel of the fungal lesion spot was then compared to the obtained calibration curve and the maximum and mean riboflavin concentrations were estimated. Three-dimensional lambda stacks were used for 3D reconstruction.

### Biological activity

Primary bat skin fibroblasts were derived from the flying membrane of a *M. myotis* specimen. Tissue was loosened mechanically with scissors without using proteases. Migratory adherent cells were cultivated for 1 month together with the remaining tissue sample. Afterwards, cells were passaged to confluency twice followed by cryopreservation of the stock in liquid nitrogen. Skin-derived cells were identified as fibroblasts based on their spindle shape combined with positive staining for the mesenchymal marker vimentin (EXBIO Praha, Vestec, Czech Republic) and the presence of typical stress-fiber organization of the actin cytoskeleton. Cell culture was negative for mycoplasma infection (MycoAlert mycoplasma detection kit, Lonza, Walkersville, MD, USA). Cells for the experiments were grown on glass coverslips in 24 well-plates (Nunc, USA) for 48 h approximately to 50% confluency and passaged no more than 10 times. Treatment with concentrations of riboflavin (dissolved directly in the cultivation medium followed by filtration with a 0.2 μm filter; Nunc) ranging from 25 to 200 μg ml^−1^ and cell culture medium control lasted for 24 h. Treated cells and controls were incubated at the end of the experiment for 20 min with MitoTracker Red CMXRos (Invitrogen, Carlsbad, CA, USA), fixed (3.7% paraformaldehyde in PBS, 20 min, room temperature), permeabilized (0.1% Triton X-100 in PBS, room temperature, 3 min), blocked (1% BSA in PBS, room temperature, 20 min) and stained with Phalloidin-Alexa Fluor 488 conjugate (Invitrogen). Staining of nuclei was performed by mounting specimens in Mowiol-DAPI (Invitrogen). Visualization was done using an inverted Olympus IX51 microscope under a 40x objective. All procedures with living cells exposed to riboflavin were performed in a dark room to prevent photooxidation damage.

### Riboflavin cytotoxicity

Quantification of cytotoxicity was based on lactate dehydrogenase activity released from damaged cells (Cytotoxicity Detection Kit^PLUS^, Roche, Mannheim, Germany). Optimum cell concentration for the assay was determined in a preliminary experiment at 15,000 cells per well. The fibroblasts were grown in Dulbecco’s modified Eagle’s high glucose medium with L-Glutamine (Biosera, Boussens, France) supplemented with 1% fetal bovine serum and grown at 37 °C in 5% CO_2_ humidified environment. Riboflavin solutions (200, 100, 50, 25, 12.5 and 0 μg ml^−1^ concentrations) were prepared as described above immediately prior to the experiment, and cells were incubated in dark at 8 °C or 37 °C for 24 h. Cytotoxicity was estimated according to the manufacturer's recommendation and recorded using ELISA reader ELx808 (BioTek, VT, USA).

### Oxidative stress quantification

Primary bat fibroblasts were grown in biological triplicates to 50% confluency in 24 well plates (Nunc), incubated with riboflavin (2 h; 200, 100, 50, 25, 12.5 and 0 μg ml^−1^ concentrations), then illuminated in the presence of ROS sensor CellROX Green Reagent (1 μM; Invitrogen) in a botanical climabox (30 min, light intensity 110 μmol of photons m^−2^ s^−1^). Cells were detached from cultivation wells with trypsin (0.1%, 200 μl per well, Sigma-Aldrich, USA). Trypsinization was stopped with complete cultivation medium (200 μl) and Hoechst 33258 solution was added (1 μM, 10 min; Invitrogen). Intensity of CellROX Green Reagent fluorescence was quantified in cell population gated as viable singlets by fluorescence-activated cell sorter LSR II (Becton Dickinson, USA).

### Histopathology and transmission electron microscopy (TEM)

Wing-membrane biopsies for standard histopathology and TEM guided by UV transillumination were fixed in 10% neutral buffered formalin and 2% glutaraldehyde, respectively. Formalin-fixed samples were embedded in paraffin, cut into 5 μm serial tissue sections and stained with periodic acid–Schiff stain. Glutaraldehyde-preserved biopsies were post-fixed in 1% OsO_4_, dehydrated in acetone, then embedded in Epon–Durcupan mixture (Epon 812, Serva, Germany; Durcupan, ACM Fluka, Switzerland). The samples were then stained with 2% uranyl acetate and 2% lead citrate and observed at 80 kV under a Philips EM 208 TEM microscope (FEI, Czech Republic).

WNS diagnosis followed guidelines issued by the USGS National Wildlife Health Center^52^. A case was positive for WNS when histologic lesions of bat WNS^10^,^13^,^14^ were present and *P. destructans* was detected.

## Additional Information

**How to cite this article**: Flieger, M. *et al*. Vitamin B_2_ as a virulence factor in *Pseudogymnoascus destructans* skin infection. *Sci. Rep.* **6**, 33200; doi: 10.1038/srep33200 (2016).

## Supplementary Material

Supplementary Video 1

Supplementary Information
