# Supplementary material for: Vitamin B2 as a virulence factor in Pseudogymnoascus destructans skin infection
Source: Sci Rep. 2016 Sep 13;6:33200. doi: 10.1038/srep33200 (PMC5020413; doi:10.1038/srep33200)
Supplement: Supplementary Information [file srep33200-s2.pdf]

Vitamin B_2 as a virulence factor in *Pseudogymnoascus destructans* skin infection

Miroslav Flieger, Hana Bandouchova, Jan Cerny, Milada Chudíčková, Miroslav Kolarik, Veronika Kovacova, Natália Martínková, Petr Novák, Ondřej Šebesta, Eva Stodůlková & Jiri Pikula

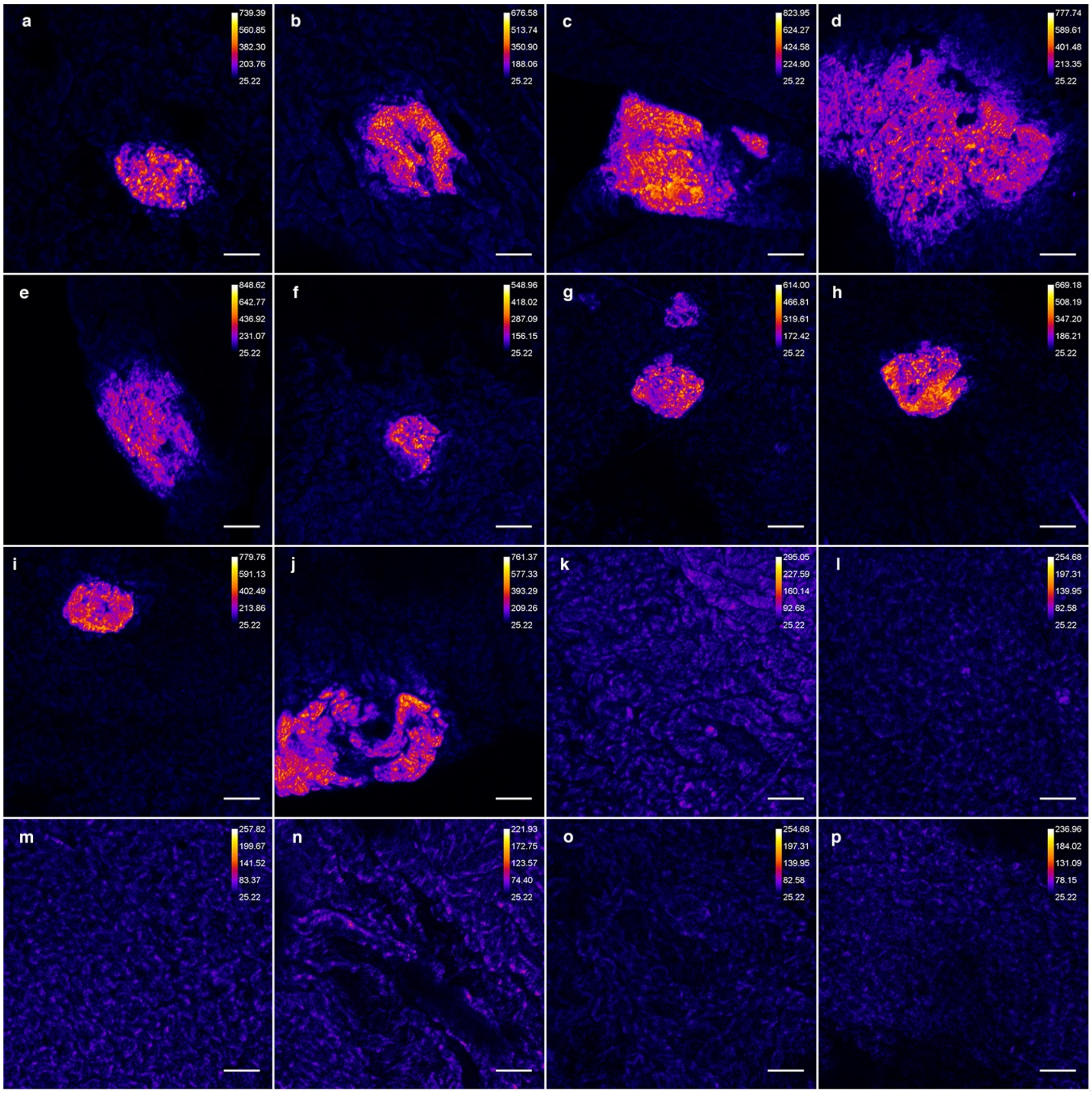

Supplemetary Fig. S1. Confocal fluorescent images of riboflavin signal in bat skin. Maximal projection of riboflavin quantification (channel 505-525 nm) in ten skin regions with WNS lesions (**a-j**) and six intact wing membranes (**k-p**). Scale bar – 50 μm.

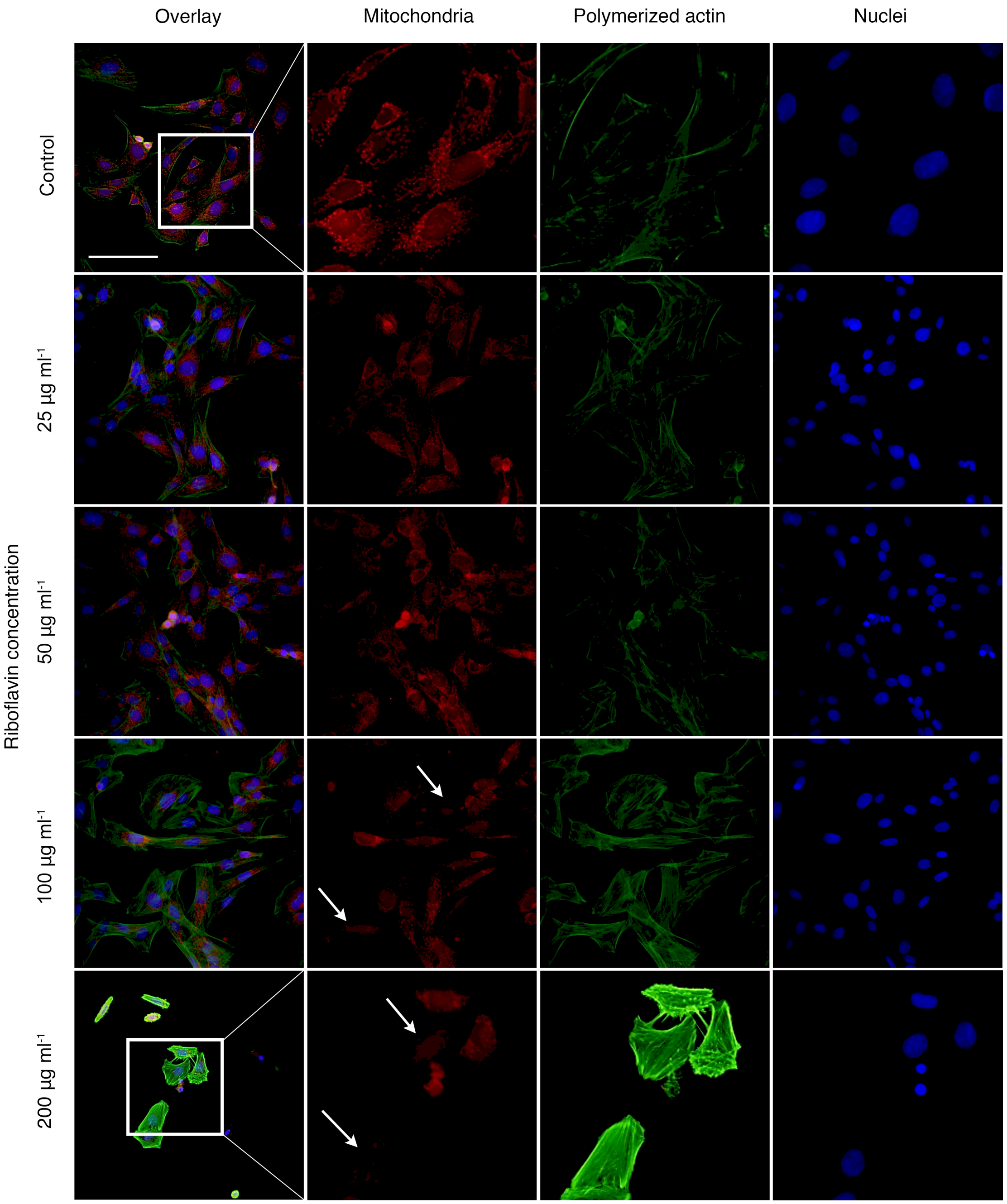

Supplementary Fig. S2. Bioactivity of soluble riboflavin in primary bat skin fibroblasts. Effect on mitochondria, actin cytoskeleton, and nuclei is shown for riboflavin concentrations ranging from 25 to 200 µg ml^{-1} and control consisting of cell culture medium. Imaging was performed using an Olympus IX51 inverted fluorescent microscope (40x). All images were acquired and processed under identical conditions. Images in the first and last row were digitally magnified from the inset window. Arrows show cells with decreased mitochondrial stainability, indicating loss of proton gradient and impairment of energy metabolism. Scale bar - 100 µm.

Supplementary Video 1. Three-dimensional lambda scan quantifying riboflavin in a WNS lesion. Fluorescence of a white-nose syndrome lesion in wing membrane of *Myotis myotis* showing elevated circumscribed dermal nodule packed with *Pseudogymnoascus destructans* hyphae producing high concentration of riboflavin. Excited with ultraviolet light (405 nm).
